# Inhibition of IL-17A Suppresses Enhanced-Tumor Growth in Low Dose Pre-Irradiated Tumor Beds

**DOI:** 10.1371/journal.pone.0106423

**Published:** 2014-09-02

**Authors:** Eun-Jung Lee, Hyo Jin Park, Ik-Jae Lee, Won Woo Kim, Sang-Jun Ha, Yang-Gun Suh, Jinsil Seong

**Affiliations:** 1 Department of Radiation Oncology, Yonsei University College of Medicine, Seoul, Korea; 2 Department of Biochemistry, Yonsei University, Seoul, Korea; Center for Cancer Research, National Cancer Institute, United States of America

## Abstract

Ionizing radiation induces modification of the tumor microenvironment such as tumor surrounding region, which is relevant to treatment outcome after radiotherapy. In this study, the effects of pre-irradiated tumor beds on the growth of subsequently implanted tumors were investigated as well as underlying mechanism. The experimental model was set up by irradiating the right thighs of C3H/HeN mice with 5 Gy, followed by the implantation of HCa-I and MIH-2. Both implanted tumors in the pre-irradiated bed showed accelerated-growth compared to the control. Tumor-infiltrated lymphocyte (TIL) levels were increased, as well as pro-tumor factors such as IL-6 and transforming growth factor-beta1 (TGF-β1) in the pre-irradiated group. In particular, the role of pro-tumor cytokine interleukin-17A (IL-17A) was investigated as a possible target mechanism because IL-6 and TGF-β are key factors in Th17 cells differentiation from naïve T cells. IL-17A expression was increased not only in tumors, but also in CD4+ T cells isolated from the tumor draining lymph nodes. The effect of IL-17A on tumor growth was confirmed by treating tumors with IL-17A antibody, which abolished the acceleration of tumor growth. These results indicate that the upregulation of IL-17A seems to be a key factor for enhancing tumor growth in pre-irradiated tumor beds.

## Introduction

In cancer radiotherapy (RT), high dose irradiated regions are always surrounded by areas of graded exposure doses ranging from medium- to low-doses [1]. Since tumor cells can be distributed at the microscopic level in a relatively wide area, circulating tumor cells might be present in surrounding areas that have received lower doses of irradiation. When tumor recurrence occurs in these low-dose irradiated areas, the recurrent tumor exhibits a more aggressive behavior than the primary counterpart [2]. However, this phenomenon has not been proved in a clinical setting yet, although the possibility that it could have a clinical implication has not been excluded. Therefore, surrounding areas receiving low-doses of irradiation, such as normal tissue in the vicinity of tumor or pre-irradiated areas, require special attention to achieve effective cancer control.

Many recent studies have shown radiation effects at lower-than-ablative doses in normal tissues. Irradiating normal tissue with low-doses could lead to the accumulation of DNA damage [3]. Chou *et al*. showed that irradiating endothelial cells with 4 Gy induced IL-6 expression, which acts through Mcl-1 expression to protect endothelial cells from irradiation-induced cell death [4]. It has been reported that irradiation-induced differential changes in the profiles of cytokines, including IL-6, IL-1α, keratinocyte-derived chemokine (KC), granulocyte colony-stimulating factor (G-CSF), and IL-17 in lung tissue irradiated with 12 Gy [5]. Several inflammatory cytokines are considered to be key factors that lead to tumor recurrence and metastasis in RT, and many recent studies have reported a role of IL-17 in tumor progression [6], [7].

IL-17, a proinflammatory cytokine that plays a critical role in the inflammatory response, autoimmune diseases, and cancer immunity, acts as a potent regulator of tumor growth as well as an important mediator in inflammatory reactions through the recruitment of monocytes and neutrophils [8], [9]. However, the role of IL-17 in tumor growth and metastasis is still unclear.

The role of pre-irradiated tumor beds on the growth of subsequently implanted tumors has been investigated extensively. Saeki *et al*. showed that pre-irradiated tumor beds that received injury induced by a single high-dose to the host vasculature and connective tissue showed impaired neovascularization in the implanted tumor [10]. However, high ablative-doses are seldom given to a substantial area of normal tissue. In current practice in stereotactic radiosurgery, where very high-dose irradiation is administered in one fraction, only a very small volume of normal tissue is included in the radiation field. Therefore, the effect of irradiating tumor beds with medium- to low-doses on the growth of subsequently implanted tumors require clinical attention and mechanism study. In the present study, we investigated the effect of medium- to low-dose pre-irradiation of tumor beds on the growth of subsequently implanted tumors. In particular, the role of the pro-inflammatory cytokine IL-17A was investigated *in vitro* and *in vivo* as a possible factor in the target mechanism.

## Materials and Methods

### Reagents

Anti-bodies of TGF-β and ROR-γ were purchased from Santa Cruz Biotechnology (CA). IL-6 and IL-17A anti-bodies were purchased from abcam (MA). p-stat-3 was purchased from Bio World (MN). IL-6 ELISA kit and CD4 anti-body were obtained from BD Bioscience (CA). TGF-β ELISA kit were purchased from BioLegend (CA). mIL-17A antibody was purchased to IL-17A neutralization from R&D system (MN). A reverse transcription system for cDNA synthesis and the primer sets of TGF-β and IL-6 were purchased from Qiagen (Hilden, Germany).

### Animal experimental design and X-ray irradiation

Five male C3H/HeN mice, 6 to 7 weeks old (Central Lab, Japan), were used per each experimental group for this study. Mice were immobilized in specially designed mice jig and the right thighs of the mice were irradiated with 5 Gy in a single fraction using an X-Rad 320 irradiator (Precision X-ray, North Branford, CT). Mice were treated 69 cm from the radiation source (SSD) with a dose rate of 150 cGy/min with 300 kVp X-rays, using 12.5 mA and a X-ray beam filter consisting of 2.0 mm Al. On day 1 and 3 after irradiation, HCa-I [11], [12] and MIH-2 [13], [14] murine hepatocarcinoma cells (1×10^6^ cells) in 100 µl phosphate-buffered saline (PBS) were injected intramuscularly into irradiated the site. Tumor volume was calculated as volume  = π/6 X ab^2^, where a is the long axis and b is the short axis of two orthogonal diameters. The maximum allowable size of tumors in mice is 20 mm in diameter according to the IACUC (Institutional Animal Care and Use Committee) guidelines of the Yonsei University Health System. After experiments, the experimental mice were sacrificed before reaching the maximum allowable size using carbon dioxide (CO_2_).

### Tumor infiltrating lymphocytes (TILs) isolation and Flow cytometry analysis

For the isolation of TILs, tumor were chopped by clipper then incubated in 1 mg/ml collagenase type IV (Worthington, Lake wood, NJ) solution containing 0.01 mg/ml DNase I (sigma, CA) at 37°C for 20 min. TILs were isolated by Percoll gradient (sigma, CA) after washing the dissociated tissues by chilled complete RPMI medium. Isolated TILs were resuspended in PBS and stained with the indicated reagents. Cells were then washed twice, fixed in 2% paraformaldehyde solution, and immediately analyzed using a FACS Canto flow cytometer (Becton Dickinson, CA).

### Analysis of gene expression

Total RNA was isolated from tumor cells using TRIzol reagent (Invitrogen Corp., Carlsbad, CA). PCR was performed using the Step One Plus (Applied Biosystems, CA) and a QuantiTect SYBR Green PCR Kit (Applied Biosystems, Warrington, UK). The amplification program consisted of 1 cycle of 95°C with a 10 min hold (hot start), followed by 35 cycles of 95°C with a 20 sec hold, 60°C with a 20 sec hold, and 72°C with a 20 sec hold. After normalization with GAPDH, the median target level of implanted tumor only and non-irradiated bed were used as calibrators.

### Immunohistochemical and immunofluorescence staining

Tumor samples were fixed in 10% formalin and were embedded in paraffin, which was cut into 5-µm-thick sections. For immunohistochemical staining, deparaffinized sections were blocked with 10% normal horse serum for 1 h and then incubated with primary antibodies against TGF-β, CD-31 and IL-17A (1∶100). The samples were incubated with biotinylated secondary antibody (DAKO code K0675; DAKO Corp., Carpinteria, CA) and peroxidase-labeled streptavidin (DAKO code K0675). Staining was developed using the 3-3 diaminobenzidine (DAB) substrate chromogen system (DAKO Corp.).

To immunofluorescence staining, deparaffinized sections were blocked with 10% normal horse serum for 1 h and then incubated with primary antibodies against TGF-β and IL-6. And then, the samples were incubated with Alexa 594-conjugated donkey anti-rabbit IgG (Invitrogen).

Co-immunofluorescence staining was also performed to assess co-localization of CD4 and Ror-γ. The sections were incubated overnight with a mixture of anti-CD4 and anti-Ror-γ in 4°C followed by washes with PBS and incubation with a mixture of Alexa 488-conjugated donkey anti-goat IgG and Alexa 594-conjugated goat anti-rat IgG (Invitrogen). The reactions were examined using an immunofluorescence microscope.

### Cytokine detection

IL-6 and IL-17A protein were measured in culture supernatants by enzyme immunometric assays (ELISA). ELISAs were performed using specific mAb pairs for the detection of both IL-6 and IL-17A. For sandwich ELISAs, purified antimouse IL-6 and IL-17A antibodies were adsorbed to capture cytokines on 96-well immunoassay plates. The culture supernatant at the appropriate dilution was incubated following the addition of biotinylated antimouse IL-6/IL-17A mAbs. Streptavidin-HRP (BD Pharmingen) was used for detection, followed by the addition of the TMB substrate reagent set (BD Pharmingen, San Diego, CA).

### Th17 cell differentiation from naïve CD4^+^ T cells

Naive CD4^+^ T cells were isolated from spleen of C3H/HeN mice by negative selection using mouse CD4^+^ T cell isolation kit (Miltenyi Biotec). Purified-naïve CD4^+^ T cells were stimulated *in vitro* with anti-CD3 (1 µg/ml) anti-CD28 (1 µg/ml) antibodies in 5 Gy irradiated- or no irradiated media. For Th17 polarizing *in vitro*, Th17 polarizing factors except TGF-β (anti-IFNγ (2 µlg/ml), anti-IL-4 (2 µlg/ml), rmIL-6 (10 ng/ml) were added to culture media. These were cultured for 4 days.

### IL-17 neutralization in vivo

Approximately 1×10^6^ HCa-I and MIH-2 were injected intramuscularly in the pre-irradiated site day 3 after the implantation site had been irradiated with 5 Gy. 100 µg α-IL-17A antibody was treated with intraperitoneally (*i.p*.) at day 3 and day 10 after inoculation of tumor cells and with intratumoral (*i.t*.) twice a week for 2 weeks after tumor formation reached 8 mm. IgG2a antibody was administered to control mice using the same method.

### Ethics statement

All procedure of animal research was provided in accordance with the Laboratory Animals Welfare Act, the Guide for the Care and Use of Laboratory Animals and the Guidelines and Policies for Rodent experiment provided by the IACUC in Yonsei University Health System (Permit Number: 2011-0114). All animal surgery was performed after euthanasia using CO^2^, and all efforts were made to minimize suffering.

### Statistical analysis

Data are expressed as means ± SEM. Comparisons among multiple groups were performed by factorial analysis of variance (ANOVA) followed by Scheffe's test or statistical comparisons between groups were made by unpaired two-sided *t*-tests. Differences of *p*<0.05 (*) and *p*<0.01 (**) were considered significant.

## Results

### The growth of implanted tumors into low-dose pre-irradiated the tumor bed was accelerated compared with non-irradiated the tumor bed

**Figure 1:**
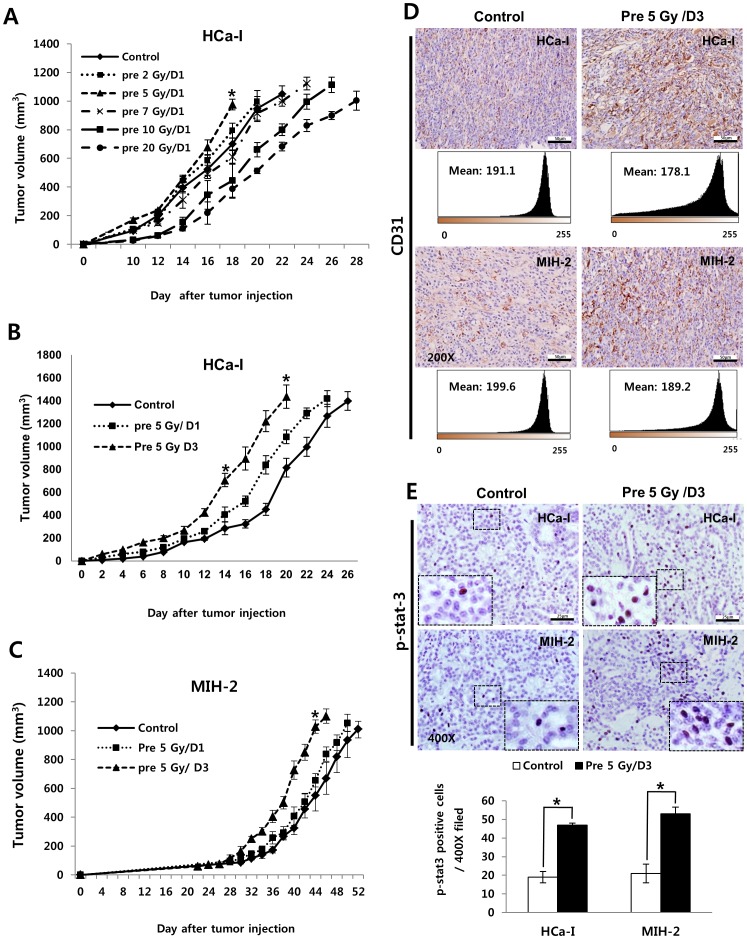
Tumor Growth and tumor related factors of implanted tumor in non-irradiated or pre-irradiated tumor beds with 5 Gy. (A) Growth rate of HCa-1 in various dose irradiated tumor beds. 1×10^6^ murine hepatocarcinoma cells (HCa-1) were injected intramuscularly in the right thigh of the mice on day 1 after the implantation site had been irradiated. (n = 5 mice per group) *p<0.05, pre 5 Gy/D1 *vs* control (ANOVA) (B) Growth rate of HCa-I and (C) MIH-2 tumors (n = 5 mice per group), (D) expression of CD31 and (E) p-stat3 in implanted tumor. Approximately 1×10^6^ murine hepatocarcinoma cells (HCa-1 and MIH-2) were injected intramuscularly in the right thigh of the mice on day 1 and 3 after the implantation site had been irradiated. Error bars denote ± SEM. *p<0.05, pre 5Gy/D3 *vs* control (ANOVA). The tumors were resected for histologic examination when the tumor volume reached 800 to 1200 mm^3^ and CD31 and p-stat3 were stained.

When patients with HCC are treated in single fraction of 20 Gy, the surrounding regions of the tumor are exposure to graded doses (Sup Fig. 1). Tumor cells are distributed at the microscopic level in the surrounding area that has received lower doses of irradiation. The tumor growth can be affected by low-dose pre-irradiation. Therefore, to investigate the effect of pre-irradiation on the growth of subsequently implanted tumors after pre-irradiating the tumor bed, experimental models were developed by irradiating the right thighs of C3H/HeN mice with various doses followed by implantation of syngeneic tumor cells of murine hepatocarcinoma (HCa-I). As shown in Fig 1, tumor growth was accelerated in areas that received below 5 Gy, while the growth of the implanted tumor into the high-dose irradiated tumor bed (more than 7 Gy) was slow (Fig 1A). Therefore, we investigated the effect of the low-dose pre-irradiating tumor bed and the change of tumor microenvironment by pre-irradiation using 5 Gy and syngeneic murine hepatocarcinomas (HCa-I and MIH-2) in this study. HCa-I is a rapidly growing tumor that is resistant to radiation, while MIH-2 is a slow-growing tumor that is sensitive to radiation in relative comparison to HCa-I. The growth of both tumors was faster in the pre-irradiated bed than that in the non-irradiated bed (Figure 1, B and C). In addition, expression of pro-tumor factors, such as platelet endothelial cell adhesion molecule (PECAM-1, CD 31) and p-stat3, increased in both tumors in the pre-irradiated beds (Figure 1D and E).

### Pre-irradiation on tumor bed increased TGF-β in implanted tumors and the tumor bed

**Figure 2:**
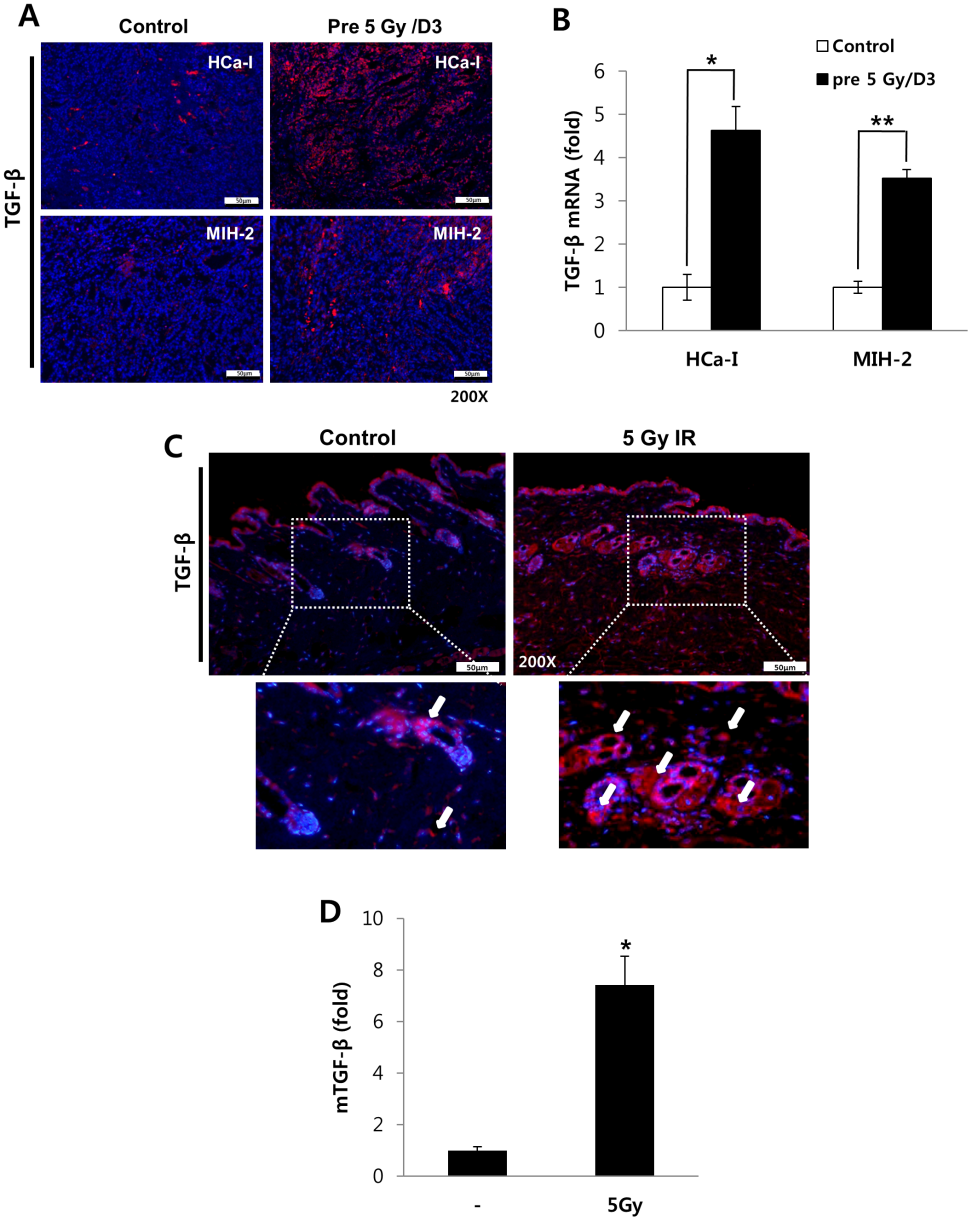
TGF-β expression. (A) TGF-β expression and (B) mRNA level in both tumors implanted into irradiated tumor beds or non-irradiated tumor beds at day 3 after irradiation. (C) TGF-β expression and (D) TGF-β mRNA level of tumor beds with or without 5 Gy-irradiation. *p*<0.05 (*) and *p*<0.01 (**) (*t*-test).

TGF-β has multiple tumor-promoting effects and it can be induced by irradiation [15]; therefore, its expression was measured in the implanted tumors. TGF-β expression and its mRNA levels were higher at the tumor edges as well as in both the tumors implanted in the pre-irradiated beds than those in the control were (Figure 2A, B and Figure S2). TGF-β expression and mRNA level in the irradiated skin and muscles of the mice on day 3 of irradiating their thighs with 5 Gy was investigated because skin and muscles acted as tumor beds. It was highly expressed in vascular endothelial cells as well as in fibroblasts of dermis (Figures 2C and D). Thus, irradiation can induce TGF-β production in a tumor and its microenvironment, which includes the stroma and vascular endothelial cells.

### Pre-irradiation on tumor bed increased TIL and IL-6 levels in implanted tumors

**Figure 3:**
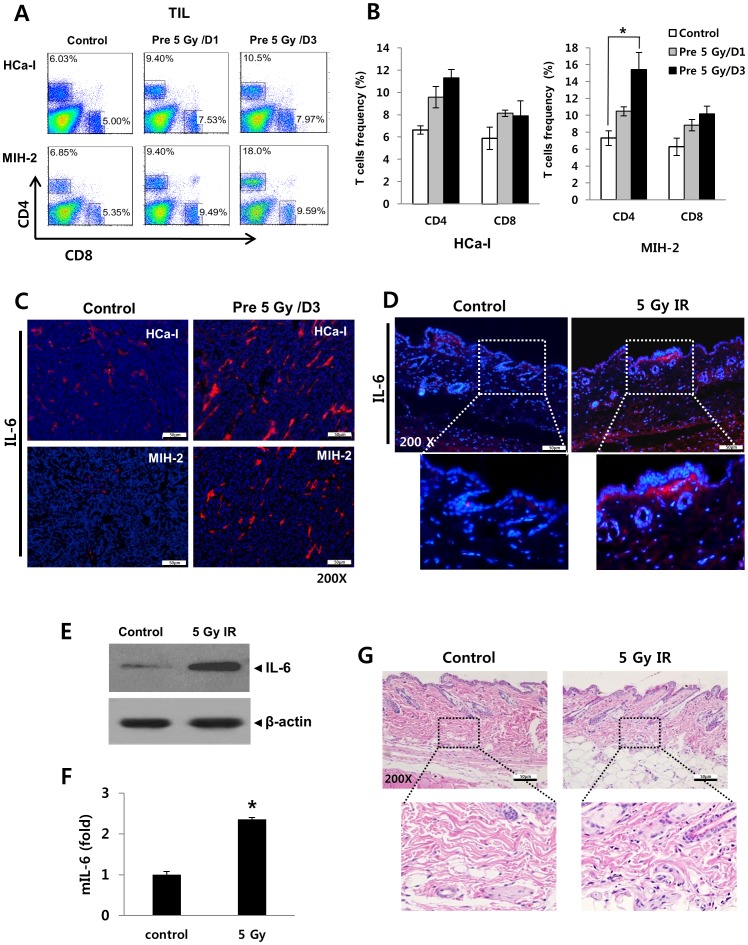
TIL frequency and IL-6 expression in implanted tumors and irradiated beds. TILs were isolated from the tumor and stained with CD4^+^ and CD8^+^ antibodies which was analyzed by FACS. (A) frequency of infiltrated CD4^+^ and CD8^+^ T cells and (B) Summary of TIL frequency in HCa-I and MIH-2 tumors. (C) IL-6 expression in both tumors. Irradiated beds were analyzed at day 3 after 5 Gy irradiation. (D) Representative immunofluorescence staining for IL-6, (E) western blotting for IL-6, (F) IL-6 mRNA level and (G) histology in 5 Gy irradiated beds. *p*<0.05 (*) and *p*<0.01 (**) (*t*-test).

IL-6 is believed to have a pro-tumor effect as well as an inflammatory effect [16]. It is secreted by immune cells and some non-immune cells such as smooth muscle cells and fibroblasts [17]. Therefore, we investigated TIL and IL-6 production in this study. TIL increased in both tumors implanted into the pre-irradiated beds compared to the control (Figure 3A and B). Similarly, IL-6 expression also increased (Figure 3C). Immunofluorescence staining was performed to check IL-6 levels of pre-irradiated tumor beds (Figure D). A 5 Gy-irradiation led to increased IL-6 levels in the tumor bed and infiltration of immune cells (Figures 3D, E, F, and G).

### Pre-irradiation on tumor bed increased IL-6 levels in the tumor-draining lymph node

**Figure 4:**
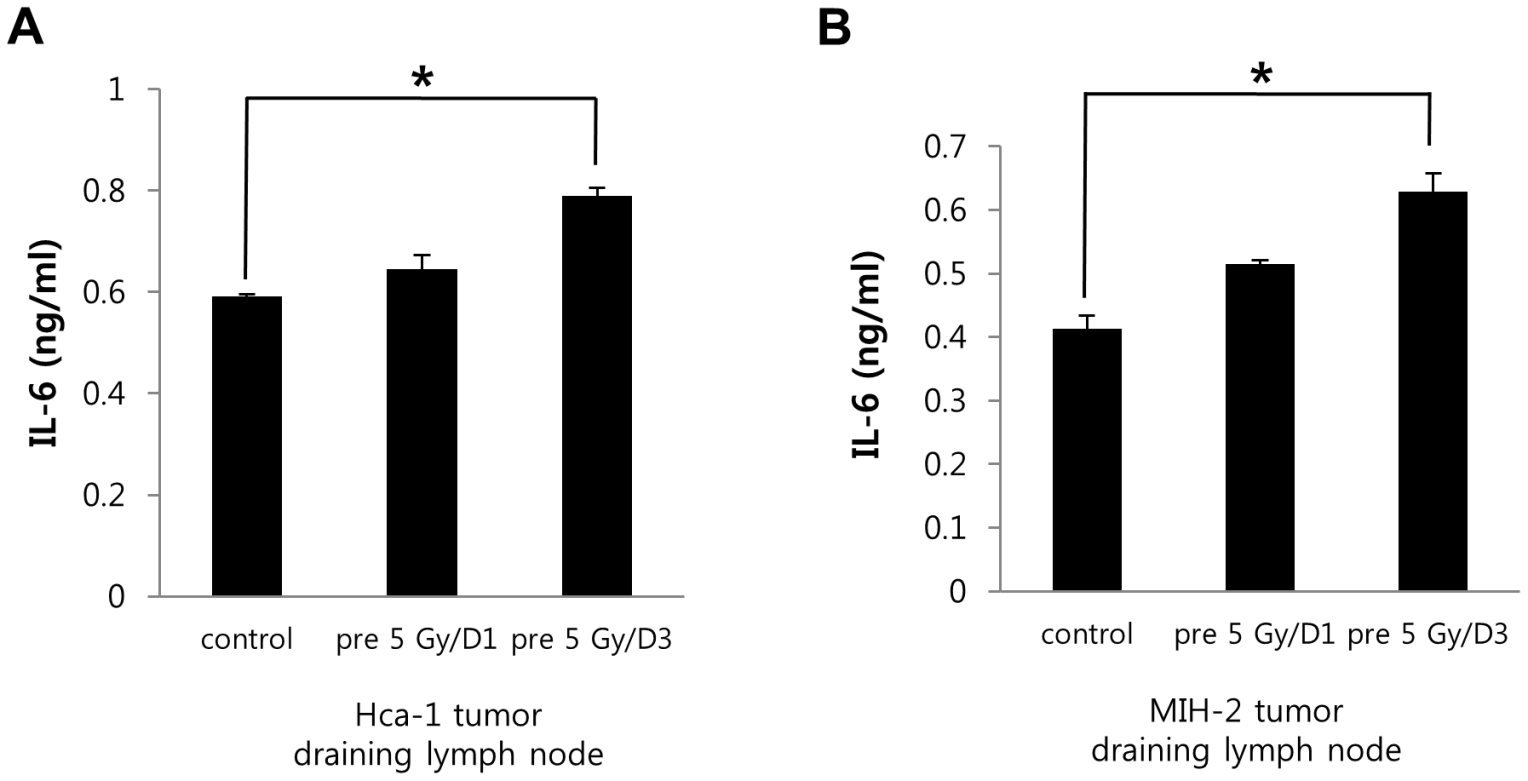
IL-6 expression in the tumor-draining lymph node of tumor bearing mice. (A and B) IL-6 expression in the tumor draining lymph node from HCa-I and MIH-2 bearing mice. *p*<0.05 (*) and *p*<0.01 (**) (*t*-test).

We next set out to assess whether pre-irradiation of tumor beds can influence IL-6 levels in tumor-draining lymph nodes, as the lymph nodes, among lymphoid tissues, show the greatest involvement in mediating inflammatory responses. As shown in Figure 4, IL-6 levels were slightly increased in the tumor-draining lymph nodes of the pre-irradiated group, compared to control. These results potentially suggest that the enhancement of tumor growth in pre-irradiated tumor beds might be influenced by factors other than IL-6.

### Pre-irradiation on tumor bed showed increased IL-17A in tumor-draining lymph node

**Figure 5:**
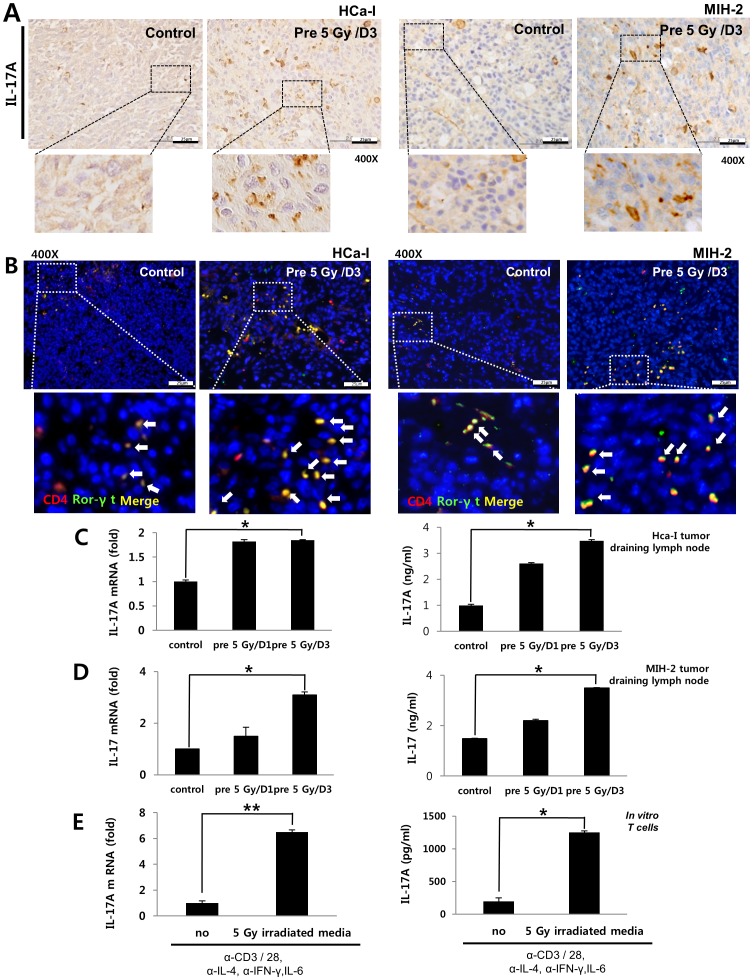
IL-17A expression in the tumor-draining lymph node of tumor bearing mice and Th17 differentiation by irradiation *in vitro*. (A) Expressions of IL-17A and (B) Th17 cells (CD4^+^ and Ror-γt double positive cells) in both tumors. (C) IL-17A mRNA level and protein expression in the tumor draining lymph node of HCa-I bearing mice. (D) IL-17A mRNA level and protein expression in the tumor-draining lymph node of MIH-2 bearing mice. (E) Th17 differentiation by irradiation *in vitro*. Murine fibroblasts were cultured for 3 days after irradiating cells with 5 Gy. Naïve CD4+ T cells were isolated from the spleen, which were then cultured for 4 days in conditioned medium derived from irradiated fibroblasts or non-irradiated fibroblasts. Secreted IL-17A from Th17 cells was analyzed by ELISA. IL-17A mRNA level was evaluated using quantitative real time PCR. rmIL-6 (10 ng/ml), α-IFN-γ (2 µg/ml), and α-IL-4 (2 µg/ml) were added to the culture media. *p*<0.05 (*) and *p*<0.01 (**) (*t*-test).

Our results showed that IL-6 and TGF-β expressions increased in tumors implanted in pre-irradiated beds. TGF-β and IL-6 are required to induce Th17 differentiation from naive T cells [18]. IL-17A, in particular, plays an important role in cancer development and in inflammatory responses [6]; therefore, we investigated IL-17A and Th17 cell populations. To investigate IL-17A and Th17 cells in tumors, both tumor tissues were stained with IL-17A antibody and co-stained with CD4 and Ror-γt antibodies because Th17 cells are CD4- and Ror-γt-positive. We found an increase in IL-17A in both tumors implanted in the pre-irradiated beds, compared to controls (Figure 5A). Th17 cells also increased in number in both tumors implanted in the pre-irradiated beds, compared to controls (Figure 5B). To evaluate IL-17A levels in T cells, CD4^+^ T cells were isolated from tumor-draining lymph nodes in mice and were cultured for 4 days in the presence of α-CD3 and α-CD28. IL-17A mRNA levels and secreted-IL-17A into the media from CD4^+^ T cells increased in the pre-irradiated group, compared to the control group (Figure 5C and D). These data show that enhanced-tumor growth is closely correlated with IL-17A in pre-irradiated tumor beds.

### Th17 cell differentiation was enhanced by irradiation in vitro

Next, to determine how irradiation to the tumor beds could induce Th17 cells, we examined Th17 cell differentiation by irradiation *in vitro*. First, we isolated dermal fibroblasts from mice and these were irradiated with 5 Gy. An irradiation of 5 Gy increased IL-6 and TGF-β levels in dermal fibroblasts (Figure S3). Isolated-naïve CD4^+^ T cells from spleen were cultured for 4 days in conditioned medium derived from irradiated-dermal fibroblasts or no irradiated-dermal fibroblasts. For Th17 polarizing *in vitro*, Th17 polarizing factors except TGF-β were added to culture media (Anti-IFN-γ (2 µlg/ml), Anti-IL-4 (2 µlg/ml) and small amount rmIL-6 (10 ng/ml) than commonly used rmIL-6 for Th17 polarization *in vitro*. As shown Figure 5E, the differentiation of naïve T cells towards Th17 cells was enhanced in the conditioned medium derived from irradiated fibroblasts.

### Tumor progression was inhibited by IL-17A neutralization in implanted tumors in pre-irradiated beds

**Figure 6:**
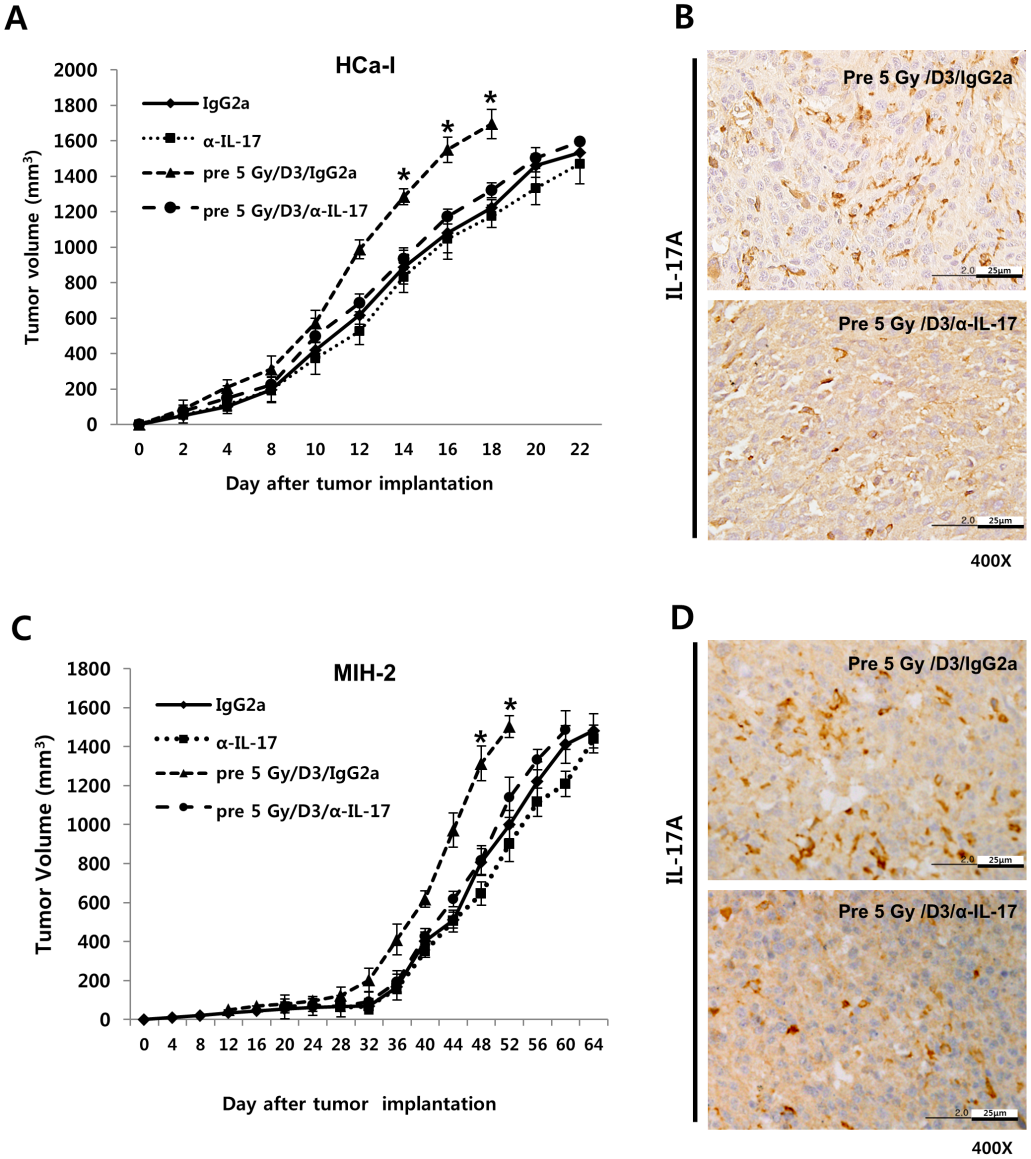
The effect of treatment with α-IL-17A neutralizing antibody on tumor growth. (A and C) Growth delay and (B and D) IL-17A expression of HCa-I and MIH-2 tumors implanted in 5 Gy irradiated beds or non-irradiated beds by α-IL-17A treatment, respectively (n = 5 mice per group). Error bars denote ± SEM. *p<0.05, pre 5 Gy/D3/α-IL-17 *vs* pre 5 Gy/D3/IgG2a (ANOVA).

When tumor cells were inoculated into irradiated tumor beds, the tumor growth rate and IL-17A expression drastically were increased relative to the controls. In addition, recombinant hIL-17A (rhIL-17A) treatment increased the proliferation of human hepatocellular carcinoma, HepG2 cells *in vitro* (Figure S4). Therefore, we further examined whether IL-17A neutralization can rescue accelerated tumor growth in pre-irradiated beds. As shown in figure 6A and C, neutralization of IL-17A suppressed the accelerated tumor growth in pre-irradiated tumor beds. Moreover, IL-17A decreased following IL-17A neutralization in both tumors implanted in the pre-irradiated beds (Figure 6B and D). Therefore, irradiating tumor beds might enhance the growth of subsequently implanted tumors via IL-17A.

## Discussion

The effect of a high, ablative-radiation dose to the tumor bed on the growth of subsequently an implanted tumor has been extensively investigated, and is known as the tumor bed effect (TBE). The TBE was first described by Frankl and Kimball and was named the TBE by Senstrom [19]. A high-dose of irradiation to the tumor bed induced injury to the host vasculature and connective tissue, resulting in impaired neovascularization in the implanted tumor [10]. It has been reported that implanted tumor growth was inhibited in high-dose (20 Gy) pre-irradiated beds, while tumor metastasis increased through increased hypoxia-associated factors [20]. Although the nature of TBE has long been recognized, its mechanism is still uncertain.

In this study, we investigated the effect of low-dose pre-irradiation (5 Gy) on the tumor bed where tumor cells were subsequently implanted. In current practice hypofractionated RT, a low-dose region usually present around the region exposed to a high, lethal dose. Tumor of areas receiving low-doses of irradiation may be affected to tumor growth by pre-irradiation. Fig 1A showed that tumor growth was accelerated in areas that received below 5 Gy. More frequently, 5 Gy-fraction hypofractionated RT is used in a clinical setting [21], [22], thus 5 Gy was used in this study.

The growth of implanted tumors in 5 Gy pre-irradiated beds was accelerated by IL-17A, compared to the controls. In HCa-I, tumor growth in the pre-irradiated beds was faster in the pre-5 Gy/D1 and D3 groups than in the control group, and was correlated with IL-17A production. However, MIH-2 showed faster growth only in the pre-5 Gy/D3 group, and there was only a slight difference in tumor growth between the control and pre-5 Gy/D1 groups. This might be attributable to a decrease in the potency of IL-17A in the pre-5 Gy/D1 group of MIH-2 than that in HCa-I.

In this study, tumor growth was suppressed by IL-17A neutralization; however, growth was still slightly faster than that in the non-irradiated control. This suggests that other factors in addition to IL-17A might promote tumor growth in pre-irradiated beds.

Several studies have shown that IL-6 inhibits radiation-induced apoptosis and enhances cell survival via stat3 activation [23], [24]. Fujikawa *et al*. reported that the IL-6-induced inflammatory response can suppress immune-mediated anti-tumor effects [25]. In this study, IL-6 level in the tumor-draining lymph nodes of irradiated group was slightly induced compared to control. Our results also showed that a 5 Gy irradiation increased TGF-β. TGF-β suppresses tumor growth during tumor initiation, but it may in fact promote tumor progression and cancer cell invasiveness in later stages [26]. Accordingly, irradiation-induced IL-6 and TGF-β could act separately as a pro-tumor factor as well as accelerated tumor growth through the induction of IL-17A in this study. However, IL-17A was significantly increased in draining lymph nodes of the irradiated group, compared to IL-6 and its expression exhibited a correlation with enhanced tumor growth. Therefore, IL-17A might be a major factor for enhancing tumor growth in low-dose pre-irradiated tumor beds.

IL-17A is mainly secreted by Th17 cells, which may influence cancer progression. IL-17B, IL-17C, IL-17D, IL-17E/IL-25, and IL-17F have all been identified in humans and cloned [27]. Among these, IL-17A has been detected in several human cancers. IL-17A promotes hepatocellular carcinoma (HCC) metastasis via matrix metalloproteinase (MMP) 2 and 9 [28]. In contrast, another study showed that endogenous IL-17 reduces tumor growth and metastasis [29]. Although, the role of IL-17 in cancer has not been fully studied, the clinical evidence of IL-17A and Th17 cells as pro-tumor factors in HCC have recently been reported. It has shown that increased IL-17 production in T cells from tumors or blood of HCC patients is correlated with both micro-vessel density and poor prognosis, suggesting the potential role of IL-17 as a pro-tumor factor [30], [31]. It was also reported that the inhibition of monocytes/macrophage-derived inflammation in hepatoma-bearing mice suppresses tumor-infiltrating Th17 cell numbers, resulting in a reduction of tumor growth [32]. Thus, IL-17A seems to be a specific target in HCC. Indeed, this study demonstrated that low-dose pre-irradiation of tumor beds subsequently enhanced implanted-hepatoma growth *in vivo*, which was suppressed by IL-17A neutralization. Therefore, we suggest that IL-17A may be used as a novel therapeutic approach to improve the outcome of patients with HCC receiving RT through preventing recurrence in low-dose normal liver. However, further studies are needed to determine whether blocking IL-17A helps in preventing recurrence of both the primary irradiated tumor and recurrence in low-dose normal livers.

In conclusion, the results of our study suggest that low-dose irradiation of tumor beds can induce IL-17A production via IL-6 and TGF-β production, and promote the growth of subsequently implanted tumors. IL-17A seems to be a key factor for enhancing tumor growth in pre-irradiated tumor beds.

## Supporting Information

Figure S1 Iso dose lines of the patient with Hepatocellular carcinoma receiving 20 Gy single fractionation RT.
(TIF)

Figure S2 Expression of TGF-β by pre-irradiation of tumor bed in tumor border and tumor. Approximately 1×10^6^ HCa-1 cells were injected intramuscularly in the right thigh of the mice on day 3 after the implantation site had been irradiated with 5 Gy.
(TIF)

Figure S3 Murine fibroblasts were isolated from mice skin that were irradiated with 5 Gy. (A) TGF-β and (B) IL-6 expression was assessed by ELISA in irradiated-murine fibroblasts cultured media 3 day after irradiation, respectively. Data are representative of three independent experiments.
(TIF)

Figure S4 Proliferation of HepG2 cells by rhIL-17. HepG2 cells were treated with rhIL-17A of concentration from 0 to 100 ng/ml. Data are representative of three independent experiments.
(TIF)

## References

[1] Purdy JA (2008) Dose to normal tissues outside the radiation therapy patient's treated volume: a review of different radiation therapy techniques. Health physics 95: 666–676. 18849701 10.1097/01.HP.0000326342.47348.06

[2] Kuonen F, Secondini C, Ruegg C (2012) Molecular pathways: emerging pathways mediating growth, invasion, and metastasis of tumors progressing in an irradiated microenvironment. Clinical cancer research 18: 5196–5202. 22730447 10.1158/1078-0432.CCR-11-1758

[3] Pogribny I, Koturbash I, Tryndyak V, Hudson D, Stevenson SM, et al (2005) Fractionated low-dose radiation exposure leads to accumulation of DNA damage and profound alterations in DNA and histone methylation in the murine thymus. Molecular cancer research 3: 553–561. 16254189 10.1158/1541-7786.MCR-05-0074

[4] Chou C, Chen S, Cheng JC (2009) Radiation-induced interleukin-6 expression through MAPK/p38/NF-kappaB signaling pathway and the resultant antiapoptotic effect on endothelial cells through Mcl-1 expression with sIL6-Ralpha. International journal of radiation oncology, biology, physics 75: 1553–1561. 10.1016/j.ijrobp.2009.08.034 19931737

[5] Ao X, Zhao L, Davis M, Lubman D, Lawrence T, et al (2009) Radiation produces differential changes in cytokine profiles in radiation lung fibrosis sensitive and resistant mice. J Hematol Oncol 2: 6. 19187543 10.1186/1756-8722-2-6 PMC2663566

[6] Numasaki M, Fukushi J, Ono M, Narula SK, Zavodny PJ, et al (2003) Interleukin-17 promotes angiogenesis and tumor growth. Blood 101: 2620–2627. 12411307 10.1182/blood-2002-05-1461

[7] Zou W, NP R (2010) Th17 cells in tumour immunity and immunotherapy. Nature Review 10: 248–256. 10.1038/nri2742 PMC3242804 20336152

[8] Park H, Li Z, Yang XO, Chang SH, Nurieva R, et al (2005) A distinct lineage of CD4 T cells regulates tissue inflammation by producing interleukin 17. Nature Immunology 6: 1133–1141. 16200068 10.1038/ni1261 PMC1618871

[9] Van den Berg WB, Miossec P (2009) IL-17 as a future therapeutic target for rheumatoid arthritis. Nature Reviews Rheumatology 5: 549–553. 19798029 10.1038/nrrheum.2009.179

[10] Saeki Y, Shimazaki S, Urano M (1971) Radiation effect on the vascularization of a C3H mouse mammary carcinoma. Microangiographic studies of the tumor in preirradiated tissue and of the recurrent tumor. Radiology 101: 175–180. 5111970 10.1148/101.1.175

[11] Milross CG, Mason KA, Hunter NR, Chung WK, Peters LJ, et al (1996) Relationship of mitotic arrest and apoptosis to antitumor effect of paclitaxel. Journal of the National Cancer Institute 88: 1308–1314. 8797771 10.1093/jnci/88.18.1308

[12] Kim W, Seong J, Oh HJ, Koom WS, Choi K, et al (2011) A novel combination treatment of armed oncolytic adenovirus expressing IL-12 and GM-CSF with radiotherapy in murine hepatocarcinoma. journal of radiation research 52: 646–654. 21952320 10.1269/jrr.10185

[13] Irie M, Homma S, Komita H, Zeniya M, Kufe D, et al (2004) Inhibition of spontaneous development of liver tumors by inoculation with dendritic cells loaded with hepatocellular carcinoma cells in C3H/HeNCRJ mice. International journal of cancer 111: 238–245. 15197777 10.1002/ijc.20247

[14] Park HJ, Kusnadi A, Lee EJ, Kim WW, Cho BC, et al (2012) Tumor-infiltrating regulatory T cells delineated by upregulation of PD-1 and inhibitory receptors. Cellular immunology 278: 76–83. 23121978 10.1016/j.cellimm.2012.07.001

[15] Maehara Y, Kakeji Y, Kabashima A, Emi Y, Watanabe A, et al (1999) Role of transforming growth factor-beta 1 in invasion and metastasis in gastric carcinoma. Journal of clinical oncology 17: 607–614. 10080606 10.1200/JCO.1999.17.2.607

[16] Hodge DR, Hurt EM, Farrar WL (2005) The role of IL-6 and STAT3 in inflammation and cancer. European journal of cancer 41: 2502–2512. 16199153 10.1016/j.ejca.2005.08.016

[17] Romano M, Sironi M, Toniatti C, Polentarutti N, Fruscella P, et al (1997) Role of IL-6 and its soluble receptor in induction of chemokines and leukocyte recruitment. Immunity 6: 315–325. 9075932 10.1016/s1074-7613(00)80334-9

[18] Morishima N, Mizoguchi I, Takeda K, Mizuguchi J, Yoshimoto T (2009) TGF-beta is necessary for induction of IL-23R and Th17 differentiation by IL-6 and IL-23. Biochemical and biophysical research communications 386: 105–110. 19501566 10.1016/j.bbrc.2009.05.140

[19] Stenstrom KW, Vermund H, Mosser DG, Marvin JF (1955) Effects of roentgen irradiation on the tumor bed. I. The inhibiting action of local pretransplantation roentgen irradiation (1500 r alpha) on the growth of mouse mammary carcinoma. Radiation Research 2: 180–191. 14372038

[20] Rofstad EK, Mathiesen B, Henriksen K, Kindem K, Galappathi K (2005) The tumor bed effect: increased metastatic dissemination from hypoxia-induced up-regulation of metastasis-promoting gene products. Cancer research 65: 2387–2396. 15781654 10.1158/0008-5472.CAN-04-3039

[21] Hasselle MD, Haraf DJ, Rusthoven KE, Golden DW, Salgia R, et al (2012) Hypofractionated image-guided radiation therapy for patients with limited volume metastatic non-small cell lung cancer. Journal of thoracic oncology 7: 376–381. 22198429 10.1097/JTO.0b013e31824166a5

[22] Bae SH, Park HC, Lim DH, Gwak GY, Choi MS, et al (2012) Salvage treatment with hypofractionated radiotherapy in patients with recurrent small hepatocellular carcinoma. International journal of radiation oncology, biology, physics 82: e603–e607. 10.1016/j.ijrobp.2011.09.053 22208963

[23] Horiguchi A, Oya M, Marumo K, Murai M (2002) STAT3, but not ERKs, mediates the IL-6-induced proliferation of renal cancer cells, ACHN and 769P. Kidney international 61: 926–938. 11849447 10.1046/j.1523-1755.2002.00206.x

[24] Miyamoto Y, Hosotani R, Doi R, Wada M, Ida J, et al (2001) Interleukin-6 inhibits radiation induced apoptosis in pancreatic cancer cells. Anticancer research 21: 2449–2456. 11724306

[25] Fujikawa K, Matsui Y, Miura K, Kobayashi T, Oka H, et al (2000) Serum immunosuppressive acidic protein and natural killer cell activity in patients with metastatic renal cell carcinoma before and after nephrectomy. The Journal of urology 164: 673–675. 10953123 10.1097/00005392-200009010-00013

[26] Basanta D, Strand DW, Lukner RB, Franco OE, Cliffel DE, et al (2009) The role of transforming growth factor-beta-mediated tumor-stroma interactions in prostate cancer progression: an integrative approach. Cancer research 69: 7111–7120. 19706777 10.1158/0008-5472.CAN-08-3957 PMC2748342

[27] Moseley TA, Haudenschild DR, Rose L, Reddi AH (2003) Interleukin-17 family and IL-17 receptors. Cytokine & growth factor reviews 14: 155–174. 12651226 10.1016/s1359-6101(03)00002-9

[28] Li J, Lau GK, Chen L, Dong S, Lan H, et al (2011) Interleukin 17A promotes hepatocellular carcinoma metastasis via NF-kB induced matrix metalloproteinases 2 and 9 expression. PLoS ONE 6: e21816–e21816. 21760911 10.1371/journal.pone.0021816 PMC3131399

[29] Kryczek I, Wei S, Szeliga W, Vatan L, Zou W (2009) Endogenous IL-17 contributes to reduced tumor growth and metastasis. Blood 114: 357–359. 19289853 10.1182/blood-2008-09-177360 PMC2714210

[30] Zhang J, Yan J, Xu J, Pang X, Chen M, et al (2009) Increased intratumoral IL-17-producing cells correlate with poor survival in hepatocellular carcinoma patients. Journal of hepatology 50: 980–989. 19329213 10.1016/j.jhep.2008.12.033

[31] Wang W, Wang Z, Liu Y, Qin Y, Shen Q (2010) Increased level of Th17 cells in peripheral blood correlates with the development of hepatocellular carcinoma. J chinese Oncol 32: 757–761. 21163066

[32] Kuang DM, Peng C, Zhao Q, Wu Y, Chen MS, et al (2010) Activated Monocytes in Peritumoral Stroma of Hepatocellular Carcinoma Promote Expansion of Memory T Helper 17 Cells. Hepatology 51: 154–164. 19902483 10.1002/hep.23291

