# Supplementary figures and images for: Inhibition of IL-17A Suppresses Enhanced-Tumor Growth in Low Dose Pre-Irradiated Tumor Beds
Source: PLoS One. 2014 Sep 2;9(9):e106423. doi: 10.1371/journal.pone.0106423 (PMC4152254; doi:10.1371/journal.pone.0106423)

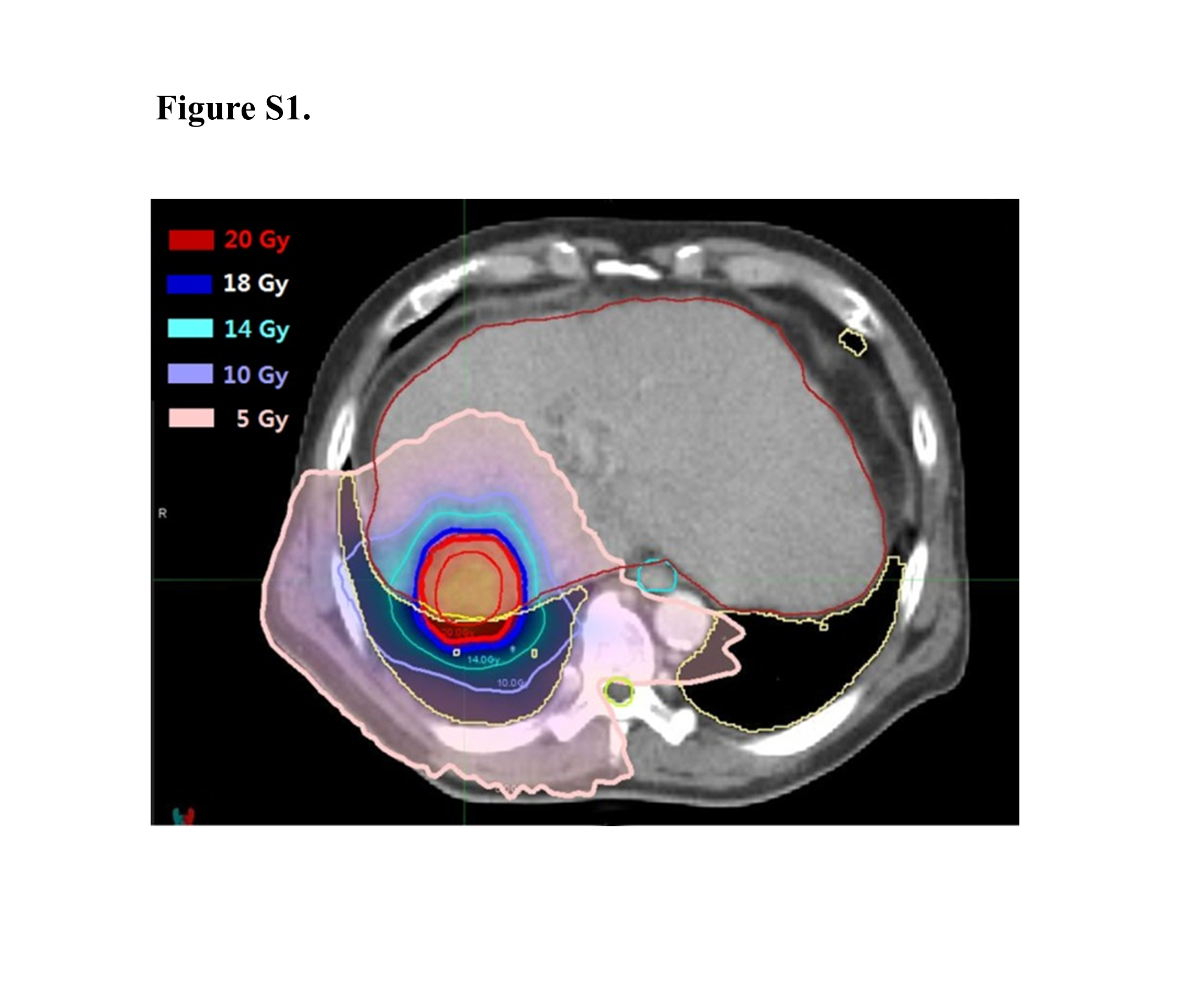

Supplement: Figure S1 — Iso dose lines of the patient with Hepatocellular carcinoma receiving 20 Gy single fractionation RT. (TIF) [file pone.0106423.s001.tif]

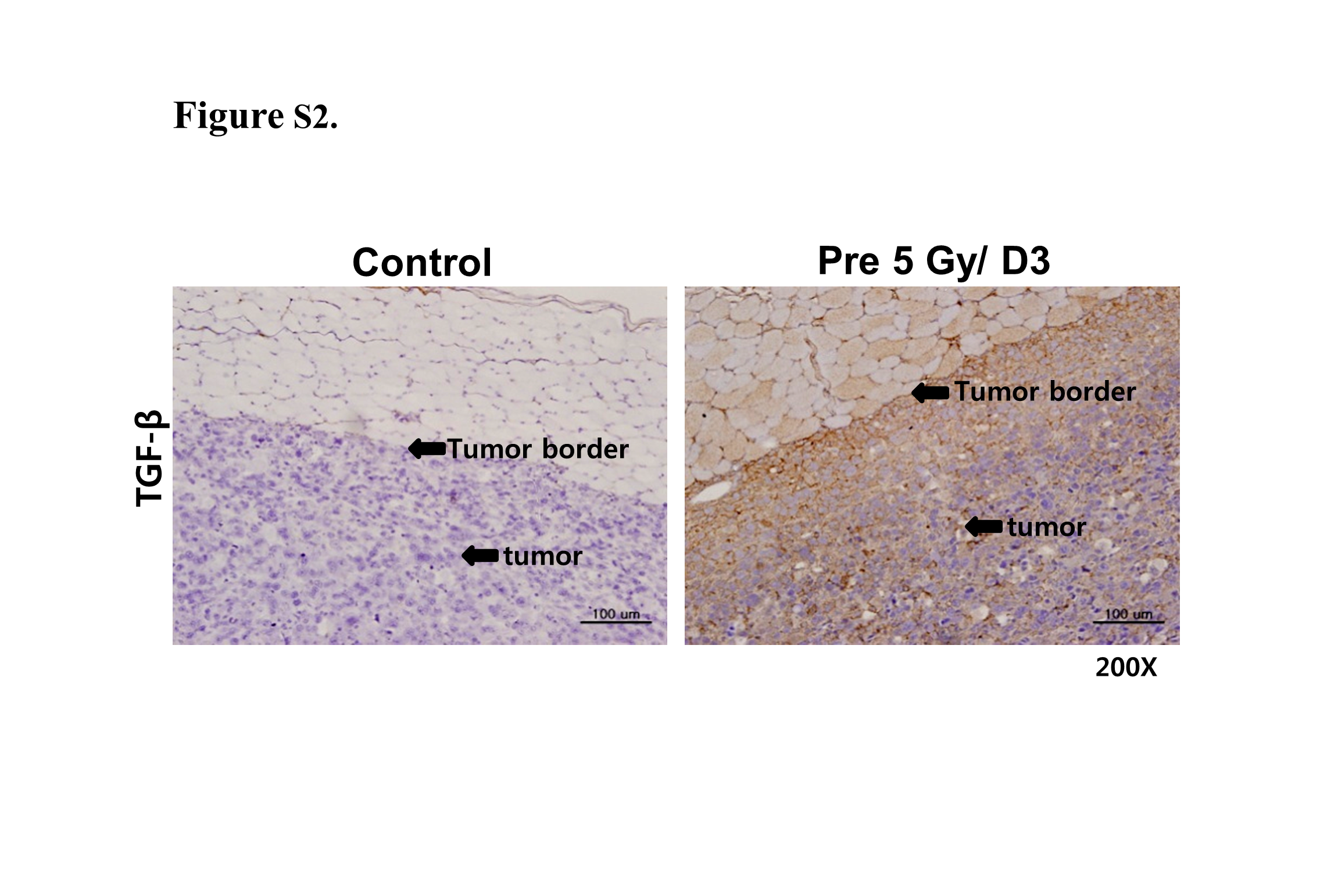

Supplement: Figure S2 — Expression of TGF-β by pre-irradiation of tumor bed in tumor border and tumor. Approximately 1×10^6^ HCa-1 cells were injected intramuscularly in the right thigh of the mice on day 3 after the implantation site had been irradiated with 5 Gy. (TIF) [file pone.0106423.s002.tif]
